# Variation in song structure does not predict associative learning performance in zebra finches (*Taeniopygia castanotis*) raised under controlled cultural conditions

**DOI:** 10.1007/s10071-026-02077-x

**Published:** 2026-06-18

**Authors:** Sébastien Derégnaucourt, Lucille Le Maguer, Nicole Geberzahn

**Affiliations:** 1 Laboratoire Éthologie Cognition Développement, Université Paris Nanterre, 200 Avenue de la République, Nanterre, Cedex F92001 France; 2 Institut Universitaire de France, Paris, 75005 France

**Keywords:** Birdsong, Vocal learning, Domain-specific cognition, Reversal learning, Acoustic communication, Individual variation

## Abstract

Vocal learning in songbirds requires the integration of auditory perception, sensorimotor learning, and performance monitoring, yet its relationship to general cognitive abilities remains unclear. We tested whether interindividual variation in song structure predicts performance in associative learning and reversal learning tasks in domesticated zebra finches (*Taeniopygia castanotis*) raised under controlled cultural conditions, in a colony founded with males trained to sing an identical song. We quantified variation in song phenotype using a composite measure summarizing motif duration, syllable number, motif elements, and similarity to the colony song model, and assessed performance in a foraging task across three phases: training, initial learning, and reversal learning. We found that this composite measure of song structure did not predict performance in any phase. Together, these results are consistent with evidence from multiple songbird species showing that individual variation in song structure is not tightly linked to individual differences in other cognitive abilities.

**Supplementary Information:**

The online version contains supplementary material available at https://doi.org/10.1007/s10071-026-02077-x.

## Introduction

Cognitive abilities have been extensively investigated in birds, both in captivity and in the wild, revealing levels of behavioral flexibility and problem-solving once thought to be restricted to mammals, particularly primates (ten Cate and Healy 2017). Among birds, songbirds have attracted particular attention because of their capacity for vocal production learning, a rare trait in the animal kingdom that has independently evolved in only a few mammalian taxa and three avian orders: *Psittaciformes*,* Trochiliformes*,* and Passeriformes* (Janik and Slater 2000; Petkov and Jarvis 2012). The convergent evolution of vocal learning across distant lineages suggests that this trait confers substantial adaptive benefits and has played a key role in the evolution of complex communication systems.

Cognition can be broadly defined as the mechanisms by which animals acquire, process, store, and act upon information from their environment, encompassing perception, learning, memory, attention, and decision-making (Shettleworth 2009). Under this definition, vocal learning in songbirds is a cognitively demanding task, as it requires the integration of sensory processing, memory formation, motor learning, and social information (Searcy and Nowicki 2019). Song learning involves a sensory phase, during which young birds memorize one or more acoustic models, and a sensorimotor phase, during which auditory feedback is used to match vocal output to the memorized template (Brainard and Doupe 2002). Notably, these phases may be separated by delays of several weeks or even months, highlighting the remarkable efficiency of auditory memory in songbirds (Marler and Peters 1982; Geberzahn et al. 2002; Geberzahn and Hultsch 2003). In many oscine species, song learning is restricted to a sensitive period early in life (“age-limited learners”), whereas in others, song remains plastic throughout adulthood (“open-ended learners”) (Brainard and Doupe 2002). The zebra finch (*Taeniopygia castanotis*) belongs to the former category. Young males acquire their song between approximately 25 and 90 days post-hatch, primarily by memorizing and imitating the song of adult conspecifics, often the father, although learning from unrelated adults and peers has also been documented (Immelmann 1969; Derégnaucourt and Gahr 2013). Once crystallized, the song remains stable throughout life. Both song memorization and production rely on a specialized network of interconnected brain nuclei, the song control system, which occupies a substantial proportion of the avian telencephalon (Nottebohm et al. 1976; Trusel et al. 2025).

Birdsong is also a culturally transmitted trait. Song learning depends on experience during development and can vary across individuals, populations, and generations, giving rise to cultural traditions and, in some species, stable geographical dialects (Catchpole and Slater 2008). Such vocal cultures influence social cohesion, mate choice, and reproductive isolation, and are increasingly recognized as having important consequences for avian evolution. Experimental work under controlled laboratory conditions has shown that population-level vocal variation can emerge through social learning alone, even in species lacking clear dialects in the wild, such as the zebra finch (Wang et al. 2022). When colonies are founded with males producing distinct song types, these patterns can be transmitted across generations, leading to the emergence of colony-specific dialects that are behaviorally meaningful to receivers. In particular, females preferentially respond to songs matching their colony’s song type, as shown in both recent studies (Le Maguer et al. 2021; Wang et al. 2022) and earlier work demonstrating learned preferences for conspecific and closely related heterospecific songs (e.g., Clayton 1990a,b). Recent studies show that vocal culture in zebra finches is shaped by a dynamic interplay between imitation and innovation. While song learning relies on the accurate copying of tutors, imitation fidelity varies across individuals and social contexts, contributing to differences in song structure (Tchernichovski and Nottebohm 1998; Holveck et al. 2008; Tchernichovski et al. 2021; see Derégnaucourt 2011 for a review; Derégnaucourt et al. 2013). In particular, pupils exposed to less diverse song models tend to introduce more improvisations, suggesting that innovation plays a compensatory role in maintaining vocal diversity within groups (Tchernichovski et al. 2021; Le Maguer et al. 2026). This balance between copying and modification may prevent both excessive convergence and uncontrolled divergence of song features. Consistent with this framework, an experimental study has shown that constraining the acoustic diversity of song models at colony foundation can give rise to distinct cultural lineages that persist over generations (Le Maguer et al. 2026). Although most individuals converge on the founding motif, song similarity gradually decreases over time as novel variants are introduced, particularly at higher levels of organization such as song bouts. Together, these findings indicate that vocal production learning can maintain diversity despite strong cultural constraints, a process that may parallel mechanisms proposed for the evolution of human language.

Birdsong serves multiple functions that vary according to socio-ecological context. At the intrasexual level, song plays a central role in territory defense and neighbour recognition, while at the intersexual level it contributes to mate attraction and stimulation. In some species, territory defense can also involve intersexual interactions, such as in duetting species (Catchpole and Slater 2008). In highly social species, song may also function as an affiliative signal, supporting group cohesion and social learning, and preferences for local song variants may reflect not only sexual selection but also social selection (Loning et al. 2023). Female birdsong has been historically underestimated, yet recent studies reveal that it is widespread, ancestral, and plays important roles in social communication, including sexual selection and social interactions (Garamzsegi et al. 2007; Odom et al. 2014; Bosca et al. 2025). Several theoretical frameworks propose that song functions as an honest signal of male quality (Gil and Gahr 2002; Searcy and Nowicki 2008). Building on the concept of a positive manifold in cognitive performance described in humans (Spearman 1904), it has been suggested that birdsong complexity or learning accuracy could reflect underlying cognitive abilities (Nowicki et al. 2000; Boogert et al. 2008; Searcy and Nowicki 2019). Thus, males producing more complex or accurate songs may not only excel at vocal learning, but also perform better in other cognitive domains, reflecting broader individual differences in cognitive abilities (e.g. Boogert et al. 2008). At least ten studies conducted across multiple songbird species (Table 1, all but two reviewed in Searcy and Nowicki 2019; Boogert et al. 2008; Boogert et al. 2011; Keagy et al. 2011; Farrell et al. 2012; Sewall et al. 2013; Templeton et al. 2014; Anderson et al. 2017; DuBois et al. 2018; Soha et al. 2019; Robayo-Noguera et al. 2025) have examined the relationship between song learning and various cognitive abilities, including associative learning, problem-solving and spatial learning. These studies report mixed results: positive correlations in some cases, negative relationships in others, and no significant association in several instances (Searcy and Nowicki 2019; Soha et al. 2019; Robayo-Noguera et al. 2025). Such variability may arise from differences in both the song traits measured and the cognitive tasks employed, suggesting that song learning and other cognitive domains, such as associative learning and problem solving, may rely on partially distinct underlying processes (Searcy and Nowicki 2019).

**Table 1:** Summary of studies examining the relationship between song traits and cognitive performance in songbirds

Study	Species	Song measures	Cognitive task	Results
Boogert et al. 2008	Zebra finch	Total number of elements per song phrase	Associative learning (foraging task)	Positive
		Number of unique elements per song phrase		
		Song phrase duration		
Templeton et al. 2014	Zebra finch	Total number of elements per song phrase	Associative learning (foraging task)	No association
		Number of unique elements per song phrase	Problem-solving (detour task)	
		Song phrase duration		
Boogert et al. 2011	Song sparrow	Song type repertoire size	Associative learning (foraging task)	Negative (foraging task) and positive (detour-reaching task)
			Inhibitory control (detour-reaching task)	
Sewall et al. 2013	Song sparrow	Song repertoire size	Spatial cognition (foraging task)	Negative
Anderson et al. 2017	Song sparrow	Proportion of notes copied from models	Associative learning (foraging task)	No correlation
		Copy accuracy	Cognitive flexibility (reversal task)	
		Song repertoire size	Inhibitory control Problem solving	
			Spatial cognition	
Soha et al. 2019	Song sparrow	Song repertoire size	Associative learning (foraging task)	Correlations not consistent over time
		Copy accuracy	Cognitive flexibility (reversal task)	
			Inhibitory control	
			Problem solving	
			Spatial cognition	
Keagy et al. 2011	Satin bowerbird	Mimetic repertoire size (number of species mimicked)	Cognitive flexibility	No association
			Motor/technical skills	
			Problem solving	
Farrell et al. 2012	European starling	Song bout duration	Problem solving with social demonstration	Positive (foraging task)
			Spatial cognition (foraging task)	
DuBois et al. 2018	Swamp sparrow	Repertoire size	Associative learning (foraging task)	No correlation
		Vocal performance (trade-off between trill rate and frequency bandwidth)	Spatial cognition	
		Song typicality (distance to cluster centroid of the population)	Inhibitory control (detour-reaching test)	
Robayo-Noguera et al. 2025	Black-capped chickadees	Song duration	Spatial cognition	No correlation
		Vocal performance	Cognitive flexibility (reversal task)	
			Long term memory	
Audet et al. 2023	21 songbird species	Vocal learning complexity (six vocal learning characteristics; used as a proxy for song complexity)	Associative learning (foraging task)	Species with morecomplex vocal learning showed better problemsolving and larger brains, but other cognitive tasks were not strongly associated with vocal learning complexity
			Cognitive flexibility (reversal task)	
			Inhibitory control (detour-reaching test)	
			Problem solving	

No study to date has examined the relationship between song learning accuracy and cognitive performance in birds reared under tightly controlled cultural conditions during the sensitive phase of vocal learning, despite growing evidence that subtle differences in imitation fidelity can shape cultural evolution and social preferences. In the present study, we addressed this gap using a colony of domesticated zebra finches founded by males trained to produce an identical song model. By tracking the cultural transmission of this song over one year, we observed marked inter-individual variation in song learning accuracy among males hatched within the colony (Le Maguer et al. 2026). We then tested whether males producing more accurate imitations of the song model also performed better in an independent foraging task, and whether commonly used acoustic song parameters predicted cognitive performance. This approach, combining tightly controlled cultural transmission with independent cognitive testing, allows us to isolate the effects of individual differences in vocal learning from variation in exposure or tutor choice. Importantly, it also enables us to test whether individual innovation tendencies are consistent across contexts, such that birds that show greater innovation in song may also perform better in the foraging task. By providing a controlled and rigorous framework, our study complements previous work conducted in more naturalistic or variable social settings and offers a stringent test of whether vocal learning accuracy can function as an honest indicator of general cognitive quality.

## Materials and methods

### Animals and housing conditions

We used 44 adult male zebra finches (age: mean ± SD, 2.86 ± 0.28 years old) from a breeding colony maintained at the animal facilities of the University Paris Nanterre (48.8903° N, 2.2067° E). Detailed descriptions of the rearing conditions of these birds are provided in Le Maguer et al. (2021, 2026). The following description summarizes the rearing conditions and procedures previously reported in these studies. As described previously, young male zebra finches acquire their song during a sensitive period early in life by listening to and imitating, with variable accuracy, the song of one or more adult males present in their environment (Derégnaucourt 2011). Ten founder males were selected based on their production of high-fidelity copies of a same song model. All selected individuals exhibited high song similarity to the model. Males were housed together in an aviary (3.18 × 3.32 × 2.84 m) with ten females that had neither prior sexual experience nor experience with male song. A few days after hatching, these females had been housed exclusively with their mothers and siblings in a breeding room and had no physical or acoustic contact with adult males until the founding of the colony. Birds were maintained under a 14:10 h light–dark cycle (lights on at 08:00 h, off at 22:00 h) at an ambient temperature of 20–22 °C. Food (a mixture of exotic seeds and egg food) and water were provided *ad libitum*. Each aviary contained 10 plastic nest boxes with coconut fibers and cotton nesting material. Nest boxes were checked daily, and nestlings were individually marked with one numbered and two colored leg bands. All individuals used in the present study were born and raised in this colony under the conditions described above.

After one year, 81 birds had hatched in the colony (50 males and 31 females). Of the 50 males, 44 were included in the present study, while 6 were excluded due to moulting or minor injuries at the time of the experiment.

### Song recording and analysis

#### Song recording

Males were individually housed in soundproof chambers (85 × 65 × 60 cm) and recorded continuously for several consecutive days. Chambers were equipped with fans providing low airflow and OSRAM DULUX lights controlled on a 14:10 light: dark cycle. Within chambers, birds were housed in cages (46 × 22 × 26 cm) with *ad libitum* access to food (seed mix, egg food for exotic finches), water, sand, and cuttlebone. To reduce the effects of social isolation, each cage was fitted with two mirrors (10 cm diameter), one on each perch. Vocal activity was recorded continuously using Sound Analysis Pro (SAP 2011) run on a DELL Optilex GX620 PC (Windows 7), with a PreSonus AudioBox recording interface (24 bit/96 kHz) connected to a Behringer C-2 microphone positioned 20 cm above the top of the cage. Perches were located 10 cm above the cage floor, ensuring that the microphone captured vocalizations from birds perched at a typical singing height. In zebra finches, directed songs (produced in the presence of a female) are shorter and more stereotyped than undirected songs (Sossinka and Böhner 1980). All analyses were based on undirected (solo) songs, which are produced more continuously and under acoustically cleaner conditions than directed courtship songs, which are often masked by cage noise generated when males display in front of females and by female calls (Sossinka and Böhner 1980; Woolley and Doupe 2008). Males may produce some directed-like song toward mirrors; however, undirected song dominates the recordings.

#### Song analysis

**Fig. 1:**
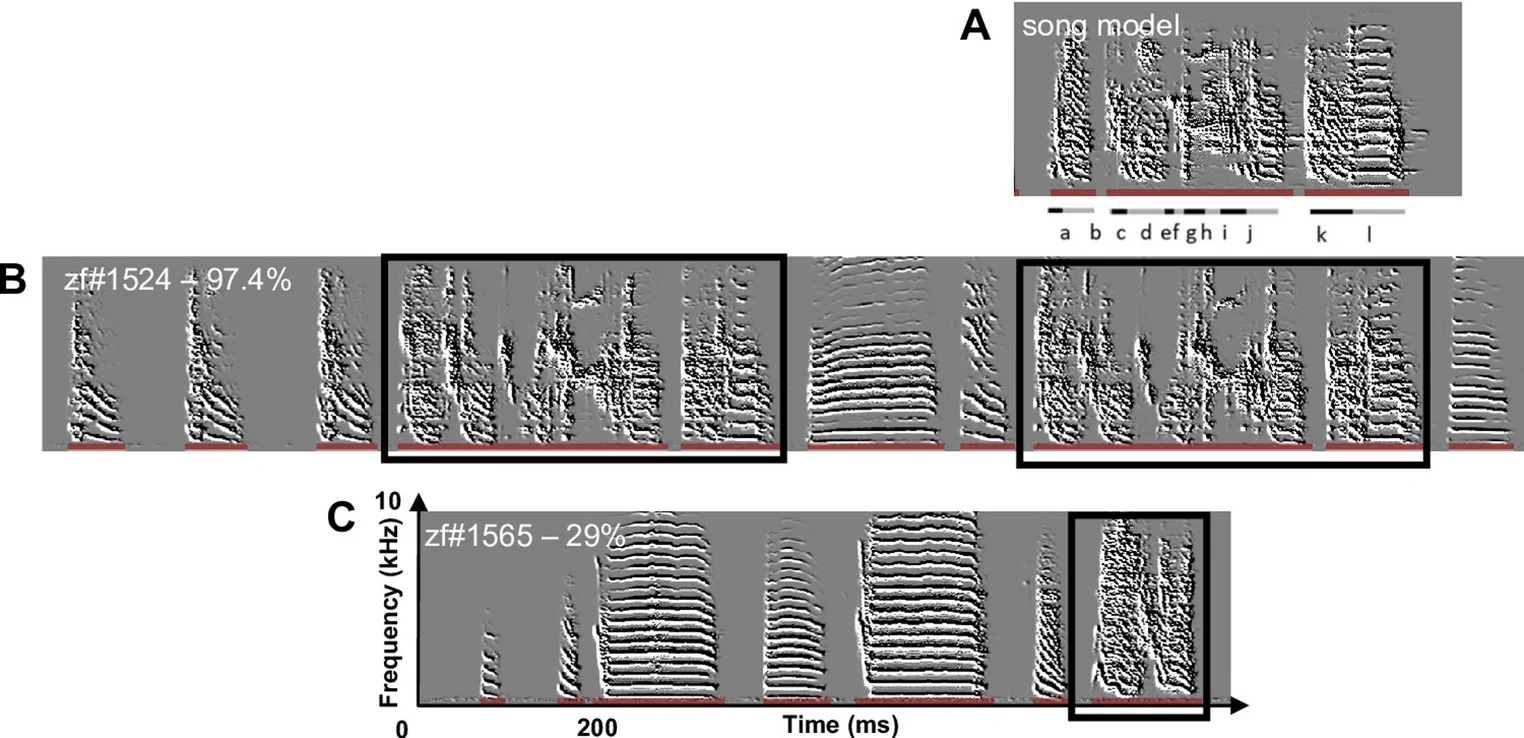
Similarity to the song model. Representative sonagrams showing (**A**) the song model, (**B**) the bird that produced the most similar rendition to the model, and (**C**) the bird with the least similar rendition. Panels B and C each show a single song bout. Motifs are highlighted with black squares, syllables are underlined in red, and individual elements - the smallest units of the song - are labeled with lowercase letters. Only the core motif, i.e., the repeated sequence consistently produced and comparable to the colony reference song, is highlighted

A zebra finch song bout consists of several introductory notes followed by one or more renditions of a motifs (Fig. 1). A motif is composed of a sequence of syllables produced in a fixed order, and each male produces a unique song motif (Derégnaucourt 2011). However, the order of syllables can vary across renditions, due to syllable deletions, insertions, alterations, or repetitions (Helekar et al. 2000). For each male, the first thirty recordings containing song from the morning of the last recording day were selected. Recordings were standardized using GoldWave (v6.36) with a 420-Hz high-pass filter and amplitude normalized to 90% to reduce potential effects of variation in amplitude on intersyllabic gaps.

##### Song analysis at the motif level: similarity to the song model and mean duration

After visual inspection of spectrograms, we selected 10 motif renditions for each bird. Selected renditions were complete, clearly identifiable motifs, produced under acoustically clean conditions, and representative of the bird’s typical singing. Renditions truncated by cage noise or artifacts were excluded. We focus our analyses on the core motif, defined here as the repeated song unit closest to the colony song model, which corresponds to the original song used to train the founder males of the colony, and corresponding to the stereotyped sequence consistently identifiable across renditions. Song learning accuracy was quantified by measuring the acoustic similarity between each male’s core motif and the colony reference song (i.e., the original song used to train the founders) using the similarity module of SAP 2011 (Tchernichovski et al. 2000). Analyses relied on asymmetric comparisons, in which the most similar sound elements of two motifs are matched independently of their temporal position. For each male, ten renditions of the core motif were extracted from .wav recordings, excluding introductory notes, and compared to the colony reference song. Similarity scores, expressed as percentages, reflect the proportion of the model’s acoustic features present in the pupil’s motif and were computed over long temporal windows (50–70 ms). The final similarity score for each individual corresponded to the mean of the ten comparisons. All offspring were evaluated relative to the same colony-level reference song, i.e., the song used to train the founders, allowing consistent assessment of song imitation across individuals and generations. This approach does not aim to identify each bird’s specific tutor, but rather to quantify how closely individual songs match the founding song tradition established in the colony. As a consequence, individuals that accurately copied a local tutor whose song had diverged from the original model may receive lower similarity scores. Thus, this measure reflects similarity to the founding song rather than tutor-specific learning accuracy. For each bird, we also measured the mean duration (in milliseconds) for the 10 selected motifs.

##### Number of elements per motif

A motif is a stereotyped sequence of syllables repeated in a fixed order, each syllable being composed of fine-grained units or “elements,” corresponding to individual vocal gestures (Fig. 1, Lachlan et al. 2016; Amador et al. 2013). For each bird, one motif was selected, and abrupt changes in amplitude and Wiener entropy (detected visually from spectrograms in SAP) were used to define “time events” marking the onset and offset of each element (Supplementary Fig. 1). This procedure allowed to calculate the number of elements per motif.

##### Number of syllable types

Songs were segmented into syllables using SAP 2011 to obtain a minimum of 300 syllables per bird for analysis. This procedure allowed the extraction 14 acoustic parameters for each unit, including duration, pitch, frequency modulation, amplitude modulation, entropy, and pitch goodness (see SAP 2011 manual: http://soundanalysispro.com/manual-1). Amplitude was excluded from subsequent analyses due to its sensitivity to recording conditions (Brumm and Slater 2006). Syllable onsets and offsets were determined via amplitude thresholding, held constant within each bird. Syllables were then clustered at the individual level using SongSeq (Daou et al. 2012), and cluster assignments were stored in Excel files. Original recordings were split into one *.wav file per syllable using a custom MATLAB script (R2017a Update 3), and syllables were organized into folders per cluster. Clustering quality was verified by visual inspection of spectrograms and auditory checks in Sound Explorer (René Jansen, University of Amsterdam) and Avisoft SASLab Pro, with manual reassignment when necessary. This analysis did permit to measure the number of different syllable types produced by each bird.

##### Song bout duration

A song bout is defined as periods of uninterrupted singing separated by silent intervals exceeding a group-specific threshold. Bout criteria were determined separately using the log survivor function of inter-song silent intervals, following standard procedures (Sibly et al. 1990; Slater and Lester 1982). Based on this approach, two bouts were considered separate when silent intervals between them exceeded 200ms. Visual inspection of spectrograms confirmed the appropriateness of this threshold for separating song bouts. We ran a MATLAB custom-made program using the pre-defined bout separation criterion to measure for each bird and for each bout the number of different syllable types and the bout duration.

### Foraging problem-solving task

Two weeks prior to the beginning of behavioral testing, birds were transferred from the aviary to cages (118 × 50 × 50 cm) located in a separate room. Birds were housed in groups of seven to eight individuals. Groups were in auditory but not visual contact with birds housed in other racks.

They were maintained under a 14:10 h light-dark cycle (lights on at 08:00 h, off at 22:00 h) at an ambient temperature of 20–22 °C, identical to the conditions in the original aviary. Food (a mixture of exotic seeds and egg food) and water were provided *ad libitum*.

To minimize handling stress, the foraging problem-solving task was conducted directly in the home cage. During testing, each bird was isolated from its cage mates by an opaque metal partition dividing the cage in half. Birds were food-deprived overnight (from 20:00 h) and tested the following morning between 10:00 and 10:30 h.

We used a plastic foraging tray (12.5 × 12.5 cm) containing 16 wells (1 cm diameter, 0.5 cm depth) arranged in a 4 × 4 grid (see Supplementary Fig. 2 for an illustration of the apparatus). This tray was familiar to the birds, as it had been present in the aviary for two months prior to the experiment and was readily used for feeding, and lids were introduced gradually during the training phase to minimize novelty-related responses. Circular plastic lids of different colors, each fitted with a vinyl insert to ensure a snug fit, were used to cover the wells; birds were not habituated to these lids prior to the experiment. During testing, perches were removed and the tray was placed on the cage floor.

The experimenter remained in the room but out of sight of the birds and simultaneously monitored different groups using a Logitech HD 1080p webcam and ContaCam video surveillance software.

The foraging problem-solving tasks consisted of three phases: a training phase followed by an initial learning phase and a reversal learning phase. Each bird underwent a maximum of eight trials per day, each lasting up to 15 min (maximum total testing time: 2 h per day). If a bird failed to feed from the tray for five consecutive trials, it was given a 15-min break during which 20 millet seeds were provided in a familiar feeder. If the bird still failed to feed, testing was postponed until the following day.

#### Training phase

The training phase consisted of five steps of increasing difficulty adapted from Brust et al. (2013, 2014) and was designed to train birds to lift the lids (Supplementary Fig. 2A). Six uniquely colored lids were used throughout training. At all training steps, lifting a lid was rewarded, as all baited wells contained food.

##### Step 1

six lids were placed adjacent to each other in one corner of the tray. Two randomly chosen wells were baited with two millet seeds each, and eight additional seeds were placed in pairs in the center of the tray.

##### Step 2

six lids were positioned in the center of the tray in two rows of three. Six randomly selected wells were baited.

##### Step 3

lids covered half of the baited wells (randomly chosen). From this step onward, each lid lifted was rewarded.

##### Step 4

lids covered almost all wells but did not fit into them.

##### Step 5

lids fully covered the wells and fit into them.

To proceed to the next step, birds had to consume at least half of the seeds in steps 1 and 2 or uncover at least half of the covered wells in steps 3 and 4. In step 5, birds had to invert at least three lids in two consecutive trials. If the experimenter was uncertain whether the criterion was met, video recordings were reviewed. Birds failing to progress after 30 trials per step were excluded from the study. Each bird could receive a maximum of 150 training trials; however, most individuals reached the learning criterion well before this limit. In total, 38 out of 44 birds reached the criterion, after 12.2 ± 6.5 [6–34] (mean ± SD, [min-max)]) trials. Three birds failed to reach the criterion in step 4 and 3 in step 5.

#### Initial and reversal learning phases

During the test phases, six lids of two different colors (three per color) were used. Lids fit into the wells, and their spatial positions were randomized between trials and between phases. Only one color was rewarded with two millet seeds per well. Rewarded colors were assigned randomly to individuals, and colors used during training were not reused. Each bird received a unique color combination. The learning test consisted of two consecutive phases: (1) *Initial learning phase*: the bird had to learn to lift the three lids of the rewarded color (Supplementary Fig. 2B); *(2) Reversal learning phase*: the previously unrewarded color became rewarded (Supplementary Fig. 2C).

Birds underwent a maximum of 30 trials per phase. To pass a phase, birds had to open the three rewarded wells first in three consecutive trials (Supplementary video 1). A bird was considered to have successfully completed the experiment if it met the criterion in the reversal learning phase.

Task performance was quantified as the number of trials required to reach criterion in each task phase (training, initial learning, and reversal learning). Birds that failed to meet this criterion within 30 trials were assigned the maximum score (30 trials) and retained in the analyses.

### Statistical analyses

All analyses were conducted in R (version 4.4.1). Acoustic song traits measured at the individual level included motif duration, number of motif elements, number of syllables, similarity to the tutor model, and song bout duration. Pairwise correlations among these traits were examined to assess collinearity. Because several acoustic variables were strongly correlated, we performed a principal component analysis (PCA) on four correlated traits (motif duration, number of motif elements, number of syllables, and similarity to the tutor model) after standardization. The first principal component (PC1) was retained as a composite measure of overall song structure. Song bout duration was only weakly correlated with the other traits and was therefore treated as an independent predictor; it was scaled prior to analysis.

Task performance was quantified as the number of trials required to reach criterion in each phase (training, initial learning, reversal learning). To retain individuals that did not reach a given phase, these cases were assigned the maximum number of trials (*n* = 30).

To test whether song characteristics predicted task performance across phases, we fitted generalized linear mixed models (GLMMs) using the glmmTMB package with a negative binomial error distribution and log link function to account for overdispersion. The main additive model included PC1, scaled song bout duration, and task phase as fixed effects, with bird identity included as a random intercept to account for repeated measures. We also tested a separate model including interactions between acoustic predictors and task phase to explore whether the effects of song structure or bout duration differed across task phases. Model selection was based on Akaike’s Information Criterion (AIC).

Additionally, a separate model including similarity to the tutor model alone was fitted to confirm whether using PC1 or this single acoustic measure (measuring the level of song imitation) affected the results. Pairwise contrasts among all task phases (training, initial learning, reversal learning) were computed using the emmeans package to allow direct comparisons, including initial vs. reversal learning.

To test the robustness of our findings, we additionally fitted generalized linear mixed models with a binomial error distribution and a logit link function, in which learning performance was coded as a binary response variable (success vs. failure to reach the learning criterion). These models included the same fixed and random effects structure as the main analyses (i.e., PC1, song bout duration, and task phase as fixed effects, and bird identity as a random intercept). This analysis was conducted to verify that the main conclusions were not dependent on the distributional assumptions or the continuous nature of the response variable.

To assess consistency in individual performance across task phases, we examined whether task performance was correlated between training, initial learning, and reversal learning phases, using Spearman’s rank correlation. We additionally tested whether the total number of elements per motif was correlated with learning speed in each task phase (training, initial learning, and reversal learning), consistent with previous studies in zebra finches using similar measures of song structure: Boogert et al. 2008; Templeton et al. 2014).

### Ethical statement

All procedures were conducted in accordance with French and European regulations for the care and use of animals in research (Directive 2010/63/EU). At the time the experiment was carried out (2015), behavioral studies involving zebra finches that did not involve invasive procedures or surgery did not require formal approval from an institutional ethical committee in France. Nevertheless, all efforts were made to minimize stress and discomfort to the birds. Housing conditions met standard welfare requirements, food and water were provided ad libitum outside controlled food deprivation periods used for behavioral testing, and experiments were conducted in the animals’ home cages whenever possible to reduce handling and disturbance. No bird was injured or showed signs of distress (e.g., feather ruffling, reduced feeding, agitation vocalizations, or escape attempts) during the study.

## Results

A principal component analysis (PCA) on motif duration, number of motif elements, number of syllables, and similarity to the tutor model showed that the first principal component (PC1) captured 73.3% of the total variance, summarizing overall song structure (Supplementary Table 1). PC1 represented a general axis of song structure, with motif duration, number of motif elements, and similarity to the song model loading negatively, whereas number of syllables loaded positively on this axis (Table 2, Supplementary Fig. 3). Song bout duration was only weakly correlated with the other traits (*r* = 0.19–0.25), so it was excluded from the PCA and treated as a separate, scaled predictor in subsequent analyses.

**Table 2:** Summary of acoustic measures included in the PCA, excluding song bout duration. For each variable, the table reports descriptive statistics and loadings on the first principal component (PC1), which explained 73.3% of the variance in song structure

Variable	Mean	SD	Min-Max	PC1 loading
Motif duration (ms)	428.6	94.2	147–592	-0.564
N Motif elements	12.9	3.4	4–18	-0.566
N song syllables	11.7	2.7	5–17	0.251
Similarity score (%)	80.6	17.6	29-97.4	-0.546

**Fig. 2:**
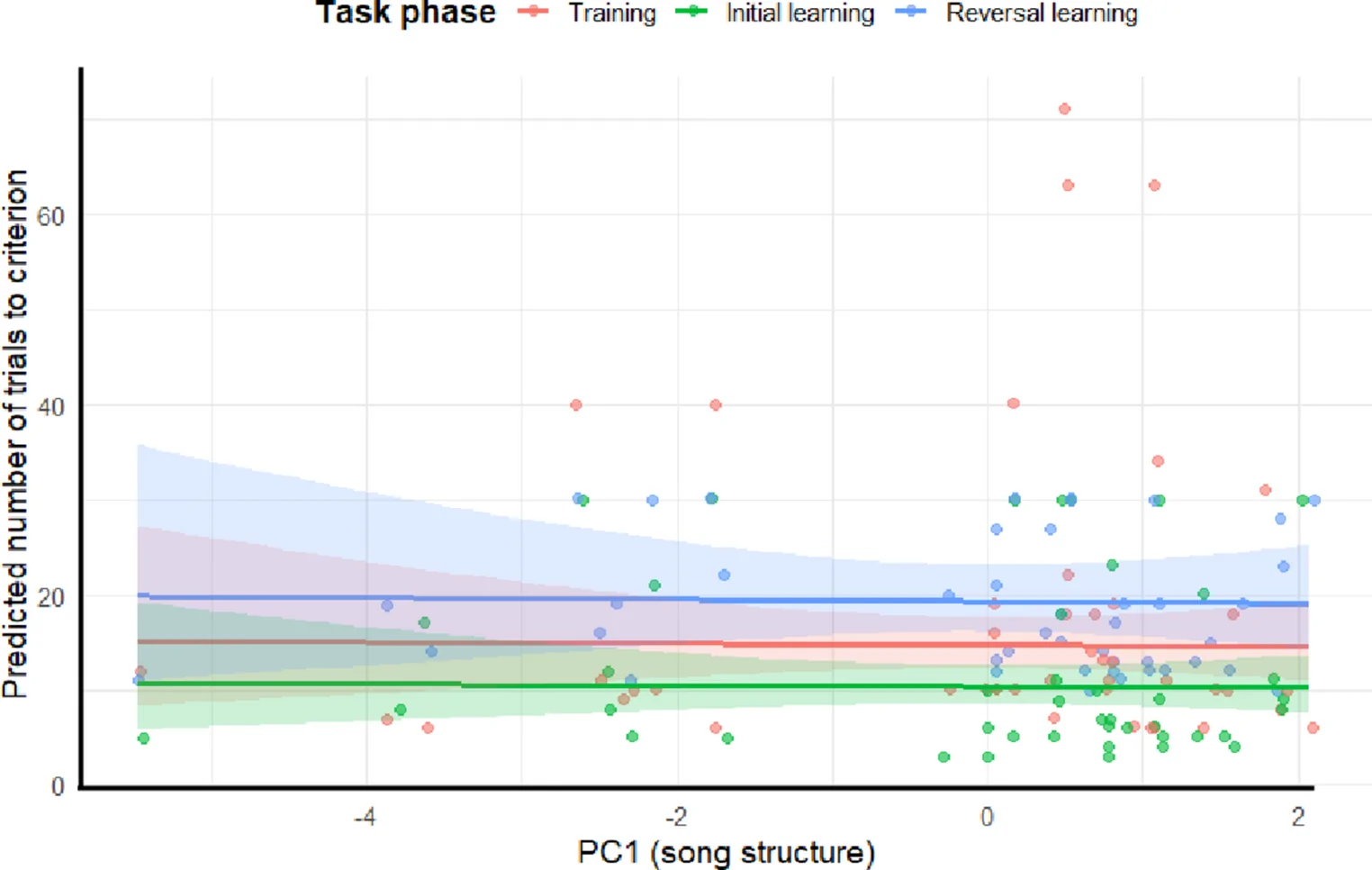
Relationship between song structure (PC1) and task performance. Predicted number of trials to reach criterion as a function of PC1, shown separately for training, initial learning, and reversal learning. Lines represent model predictions and shaded areas indicate 95% confidence intervals. PC1 did not significantly predict performance in any phase, and no interaction between PC1 and task phase was detected

Neither PC1 nor scaled song bout duration significantly predicted performance (Fig. 2; Table 3, *n* = 132 observations from 44 birds). There was no evidence that adding interactions between acoustic predictors and task phase improved model fit (Table 3; additive vs. interaction model comparison: ΔAIC = 4.7).

**Table 3:** Results of the generalized linear mixed model (GLMM; negative binomial distribution) testing the effects of acoustic structure (PC1), song bout duration, and task phase on the number of trials required. Bird identity was included as a random intercept to account for repeated measures. PC1 represents the main axis of variation in acoustic traits derived from a principal component analysis. Task phase included three levels (training, learning, reversal), with training as the reference category. Estimates are presented on the log scale. An alternative model including interactions between acoustic predictors and task phase was also tested but received less support (AIC = 919.9) than the additive model (AIC = 915.2; ΔAIC = 4.7)

Predictor	Estimate	SE	z	*p*
(intercept)	2.726	0.093	29.29	< 0.001
PC1	0.0046	0.0468	0.098	0.922
Song bout duration (scaled)	0.00157	0.0808	0.019	0.985
Learning phase	-0.385	0.0958	-4.021	< 0.001
Reversal phase	0.165	0.0923	1.793	0.073

Task phase had a significant effect on performance (*n* = 132 observations from 44 birds), with fewer trials required during initial learning compared to training and a trend toward higher trial numbers during reversal learning (Table 3).

To assess the robustness of these results, we repeated the analyses using a binary response variable (success vs. failure to reach criterion). Consistent with the main analyses, neither PC1, song bout duration, nor similarity to the song model predicted learning success. These results confirm that our conclusions are not dependent on the choice of response variable (supplementary Table 2).

To further verify that our results were not dependent on the specific representation of song structure, we additionally fitted a model including similarity to the song model as the sole acoustic predictor. This model also did not predict performance, consistent with results obtained using the composite PC1 measure (Table 4, *n* = 132 observations from 44 birds). Model comparison based on AIC indicated that the additive model (AIC = 915.23) was better supported than the interaction model (AIC = 919.93, ΔAIC = 4.7), further supporting the conclusion that acoustic traits did not differentially affect performance across phases.

**Table 4:** Results of the generalized linear mixed model (GLMM; negative binomial distribution) testing the effect of similarity to the tutor model on task performance. The model included task phase as a fixed effect and bird identity as a random intercept. The training phase was used as the reference category. Estimates are presented on the log scale

Predictor	Estimate	SE	z	*p*
(intercept)	2.783	0.365	7.62	< 0.001
Similarity to model	− 0.001	0.004	-0.16	0.87
Learning phase	-0.386	0.096	-4.03	< 0.001
Reversal phase	0.165	0.092	1.79	0.074

**Fig. 3:**
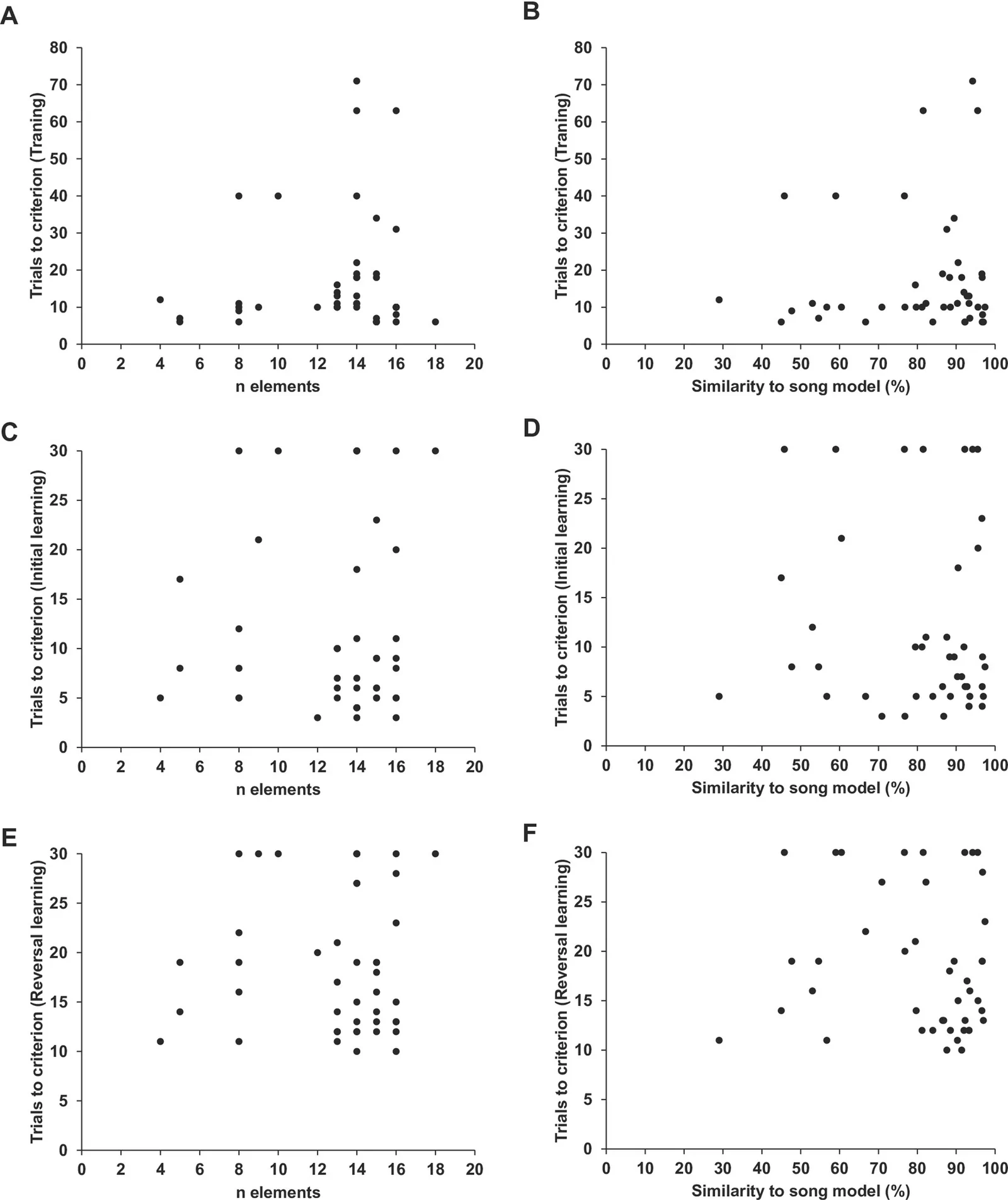
Song structure is not associated with learning performance in zebra finches. Panels A, C and E show correlations between total number of elements per motif and learning speed during training (**A**), initial learning (**C**), and reversal learning (**E**).Panels B, D and F show correlations between similarity to the song model and learning speed during training (**B**), initial learning (**D**), and reversal learning (**F**). No significant correlations were observed

We examined whether the similarity to the song model correlated with learning speed in the foraging task. No significant associations were found (Fig. 3, *n* = 44, training: Spearman’s ρ = 0.0045, *p* = 0.977; initial learning: Spearman’s ρ = -0.0746, *p* = 0.63; reversal learning: Spearman’s ρ = -0.061, *p* = 0.694). We also examined whether the total number of elements per motif correlated with learning speed in the foraging task. No significant associations were found (Fig. 3, *n* = 44, training: Spearman’s ρ = -0.0392, *p* = 0.8; initial learning: Spearman’s ρ = 0.0053, *p* = 0.973; reversal learning: Spearman’s ρ = -0.0028, *p* = 0.986).

**Fig. 4:**
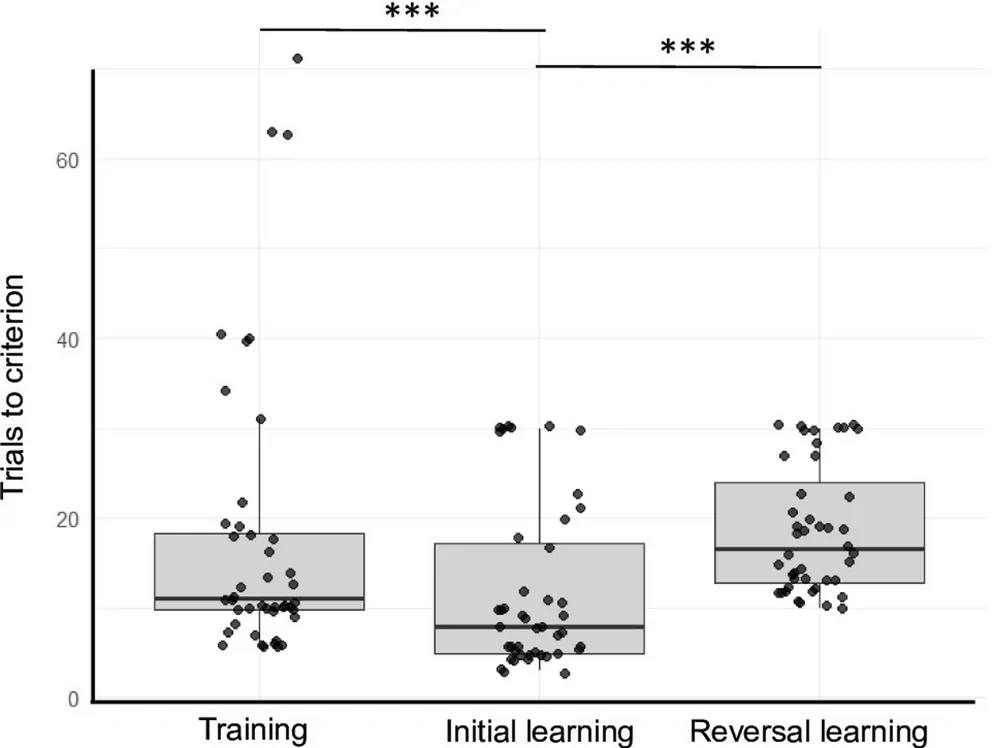
Task performance across phases. Number of trials to reach criterion during training, initial learning, and reversal learning. Points show individual birds (*n* = 44); boxes or estimated marginal means ± 95% CI summarize group performance. Birds learned faster in the initial phase than in training (*p* < 0.001) and required significantly more trials in reversal than in initial learning (*p* < 0.001), with a trend toward more trials in reversal than training (*p* = 0.073), reflecting increasing task difficulty

Task phase significantly influenced performance according to the GLMM (Table 3). Birds required fewer trials during initial learning compared to training, whereas reversal learning tended to require more trials than training, reflecting increasing task difficulty (Fig. 4; initial learning vs. training: estimate = -0.385, SE = 0.096, z = -4.02, *p* < 0.001; reversal vs. training: estimate = 0.165, SE = 0.092, z = 1.79, *p* = 0.073). A direct comparison between initial learning and reversal learning, derived from emmeans contrasts, indicated that initial learning required significantly fewer trials than reversal learning (*n* = 44, estimate = -0.550, SE = 0.096, z = -5.76, *p* < 0.001).

**Fig. 5:**
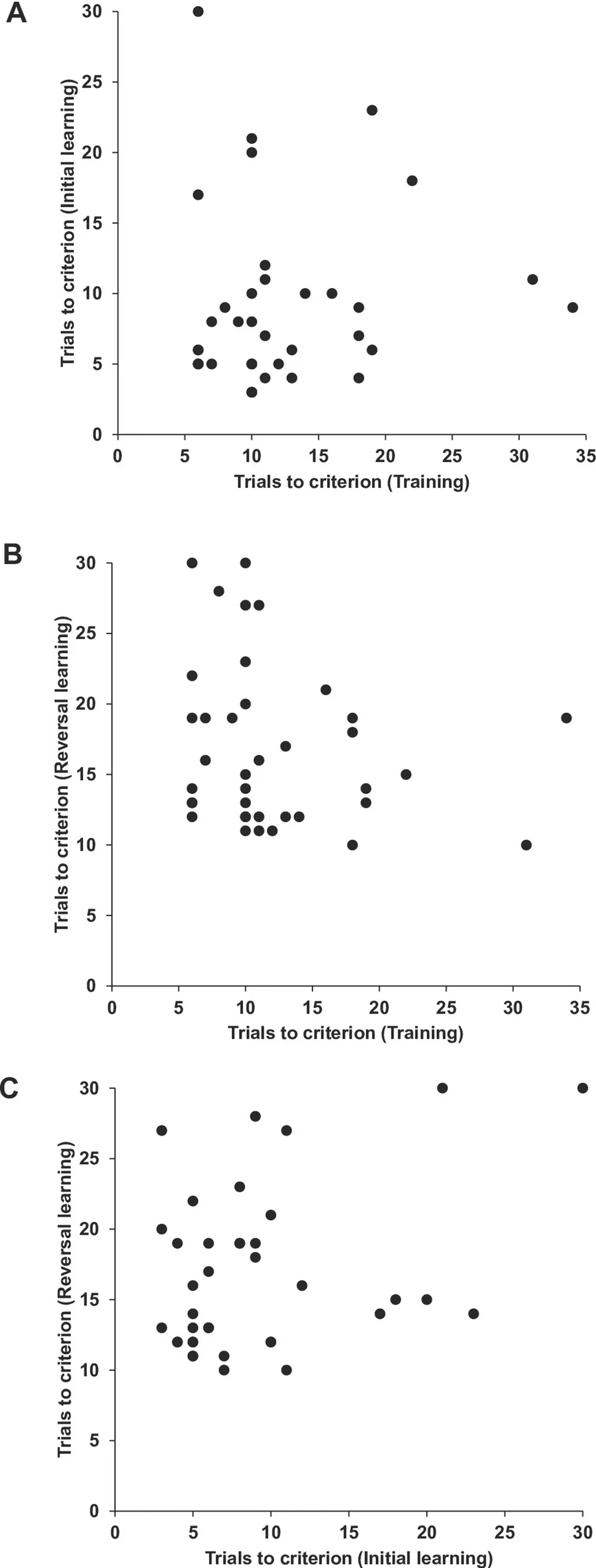
Relationships between learning phases in zebra finches. Correlations between performance in different phases of the foraging task (*n* = 38). Panel **A** shows the relationship between training and initial learning performance, Panel **B** shows the relationship between training and reversal learning performance and Panel **C**. shows the relationship between initial and reversal learning performance. No significant correlations were observed

Performance was not significantly correlated between any of the task phases (Fig. 5; training vs. initial learning: *n* = 38, Spearman’s ρ = 0.1716, *p* = 0.3; training vs. reversal learning: ρ = -0.2479, *p* = 0.134; initial learning vs. reversal learning: ρ = 0.216, *p* = 0.192).

## Discussion

The present study tested whether individual variation in song structure predicts performance in non-vocal learning tasks in zebra finches raised under controlled cultural conditions. Individual variation in song structure may reflect domain-general cognitive abilities, such that males excelling in song learning also perform better in other cognitive domains. This hypothesis is supported by some studies showing correlations between song complexity and non-vocal learning performance in passerines (Nowicki et al. 2000; Boogert et al. 2008). Our PCA indicated that motif duration, number of motif elements, similarity to the song model, and number of syllables all contributed to the first principal component (PC1), which explained 73.3% of the variance in song structure. Motif duration, number of motif elements, and similarity to the song model showed strong loadings of similar magnitude on PC1, whereas number of syllables contributed more weakly and in the opposite direction. PC1 therefore captured a major axis of variation in song structure, reflecting covariation among these acoustic traits. Despite summarizing these aspects of song quality, PC1 did not predict performance in any of the learning tasks. Most previous studies investigating links between song and cognition used measures of song complexity, such as the number of syllables or elements, as proxies for song learning ability (Boogert et al. 2008; Templeton et al. 2014). In order to improve comparability, we examined whether the total number of elements or syllables per motif correlated with learning speed in the foraging task. No significant associations were found. These results support our conclusion that variation in adult song structure does not predict performance in non-vocal learning tasks. This suggests that overall song structure, while variable among individuals, does not appear to reflect general learning ability in non-vocal domains. The weak correlations between song bout duration and the other song traits further indicate that different components of song may be influenced by largely independent factors, highlighting the multidimensional nature of song learning. Together, these results indicate that variation in adult song structure does not predict performance in non-vocal learning tasks in the zebra finch.

To ensure that this conclusion was not dependent on the statistical treatment of the response variable, we additionally re-analysed the data using a binary outcome (success vs. failure to reach criterion). These analyses yielded results fully consistent with the main models based on learning speed, with no significant effects of PC1, song bout duration, or similarity to the song model. This convergence across analytical approaches strengthens the robustness of our main conclusion that song structure is not associated with cognitive performance in this task.

A recent cross-species study by Audet et al. (2023) further explored these relationships in 214 individuals from 23 species, including zebra finches. Birds were tested in detour, associative/reversal learning, and problem-solving tasks, and a vocal learning complexity score was computed based on repertoire size, heterospecific imitation, and lifelong learning capacity. Audet et al. (2023) found a positive correlation between this vocal learning score and problem-solving performance, while no correlation emerged for other cognitive measures. Species with higher vocal learning scores also had relatively larger brains. These results suggest a coevolution of vocal learning, certain cognitive abilities, and brain size at the species level. Taken together, our results, alongside those of Audet et al. (2023), suggest that relationships between song learning and cognition may be context-dependent. In particular, different song traits may relate to distinct cognitive measures, rather than consistently predicting general problem-solving performance. The mismatch between the cognitive demands of our tasks and those underlying song learning likely contributes to the dissociation observed. Standard associative learning and reversal paradigms primarily involve visual discrimination and response updating, whereas song learning relies on auditory–motor integration and feedback-based adjustment (Brainard and Doupe 2000; Konishi 2004; Qi et al. 2025). This highlights the need for task selection that closely matches the cognitive domain of interest. Overall, in line with previous work in zebra finches reporting weak or inconsistent relationships between song learning and cognitive performance at the individual level (e.g. Boogert et al. 2008; Templeton et al. 2014), our results suggest that associations between song and cognition remain inconsistent at the within-species level and may depend on the cognitive domain considered. Importantly, a study directly addressing the cognitive capacity hypothesis in zebra finches in a more social and ecologically relevant context failed to replicate the positive association between song complexity and problem-solving performance reported by Boogert et al. (2008), finding no relationship between song complexity and performance across foraging tasks performed in flocks (Templeton et al. 2014). These authors suggested that previously reported associations may be influenced by experimental context, including stress or motivational differences associated with social isolation during testing. Overall, these findings indicate that our results are consistent with the broader pattern observed in zebra finches, where associations between song structure and cognitive performance are inconsistent, and dependent on experimental context.

In songbirds, vocal learning depends on dedicated neural circuits supporting auditory–motor integration and feedback-based refinement during a sensitive developmental period (Konishi 2004; Mooney 2022). Variation in song learning accuracy is therefore more likely to reflect differences in how conserved song learning mechanisms are functionally expressed and tuned across species (e.g., in learning precision or memory constraints), rather than differences in domain-general intelligence or behavioral flexibility. In our experimental procedure, we reduced the diversity of available song models during the foundation of laboratory colonies (Le Maguer et al. 2026). By training founder males to produce the same motif, subsequent generations largely converged on this founding motif, while introducing innovations primarily at higher levels of song organization. One limitation of our approach is that song similarity was computed relative to the original founder song rather than to each individual’s actual tutor. In a socially complex environment, juveniles may copy different males, and those tutors may themselves deviate from the original model. As a result, some individuals may produce accurate copies of their tutor’s song yet receive lower similarity scores relative to the reference song. Therefore, our measure captures fidelity to a culturally transmitted song tradition rather than tutor-specific learning accuracy. In this context, our approach aligns with our goal of assessing song variation within a controlled cultural system, where the founding song provides a common reference across individuals. Using this colony-level reference allows consistent comparisons between birds while still capturing meaningful inter-individual variation in song traits. Importantly, despite this constrained cultural input, substantial inter-individual variability rapidly emerged at multiple organizational levels, including motif–connector sequences - the sequences of call-like syllables linking motifs within a song bout - and bout structure itself (Le Maguer et al. 2026). Therefore, although some song traits exhibited reduced variation relative to the original model, the absence of correlations with learning performance cannot be fully attributed to a floor effect. Taken together, these observations suggest that vocal production learning may operate differently across hierarchical levels of song structure, and that cultural transmission can maintain polymorphism even under strong constraints. In this context, individual differences in adult song may reflect how birds reproduce and navigate culturally transmitted vocal norms rather than their general capacity to learn or behave flexibly.

These findings were also robust to an alternative binary formulation of learning performance (success vs. failure), which yielded the same qualitative pattern of results. Beyond cognition narrowly defined, sexual, social and motivational factors likely play a critical role in shaping performance in the cognitive tasks used in this study. Artificially induced song dialects influence female preferences, with females preferentially responding to songs corresponding to their colony’s song type over unfamiliar dialects (Le Maguer et al. 2021). In parallel, dopaminergic signaling within the song control system encodes performance-related feedback and drives improvements in song production (Gadagkar et al. 2016; Hisey et al. 2018), linking variation in song accuracy to reinforcement and motivation rather than general intelligence. In addition, individual variation in performance in the cognitive tasks may partly reflect differences in motivation, exploration, or reward sensitivity rather than cognitive ability per se. Individuals may differ in their willingness to engage with the apparatus, persistence after failure, or responsiveness to food rewards, all of which can affect performance in associative and reversal learning tasks. Such variation could obscure potential relationships between song learning accuracy and cognitive performance. Therefore, the absence of correlation observed here should be interpreted with caution, as it may reflect a combination of domain-specific cognitive processes and variation in motivational and social factors. Importantly, the consistency of results across both continuous (learning speed) and binary (learning success) measures indicates that our findings are not an artefact of the specific way in which learning performance was quantified.

In conclusion, our results do not provide evidence for a general view of cognition in zebra finches. Instead, they indicate that vocal learning constitutes a specialized cognitive dimension shaped by neural constraints, social experience, and reinforcement mechanisms. Notably, even under experimentally constrained cultural input, substantial inter-individual variation emerged, indicating that cognitive processes involved in song learning operate within and are influenced by socially transmitted song norms. More broadly, when considered alongside cross-species analyses (Audet et al. 2023) and within-species studies of specific song traits (Farrell et al. 2012), these findings indicate that vocal learning may coevolve with certain cognitive abilities, but within-species variation in song structure does not necessarily reflect general cognitive performance.

## Supplementary Information

Below is the link to the electronic supplementary material.


Supplementary Material 1



Supplementary Video 1


## Data Availability

data (excel file, script used for the statistical analysis) will be provided upon request.
