# Supplementary material for: Variation in song structure does not predict associative learning performance in zebra finches (Taeniopygia castanotis) raised under controlled cultural conditions
Source: Anim Cogn. 2026 Jun 18;29(1):61. doi: 10.1007/s10071-026-02077-x (PMC13529891; doi:10.1007/s10071-026-02077-x)
Supplement: Supplementary file 1 [file 10071_2026_2077_MOESM1_ESM.docx]

**Supplementary Information.** Three figures and one table.

**Supplementary figure 1. Time events mark the onset and offset of elements.** Above, a song bout (consisting of syllables, underlined in red). The inset below provides an enlarged view of a single syllable, illustrating its segmentation into elements delimited by time events. Cursive letters (a–c) label the individual elements, and blue arrows indicate distinct time events defined by abrupt changes in amplitude and Wiener entropy. In the inset, the red curve represents the amplitude, and the yellow curve represents the Wiener entropy.

**Supplementary figure 2. Foraging problem-solving task. A**: training phase. Step 1: six lids were placed adjacent to each other in one corner of the tray. Two randomly chosen wells were baited with two millet seeds each, and eight additional seeds were placed in pairs in the center of the tray. Step 2: six lids were positioned in the center of the tray in two rows of three. Six randomly selected wells were baited. Step 3: lids covered half of the baited wells (randomly chosen). From this step onward, each lid lifted was rewarded. Step 4: lids covered almost all wells but did not fit into them. Step 5: lids fully covered the wells and fit into them. B: Initial learning phase: the bird had to learn to lift the three lids of the rewarded color. C: reversal learning phase: the previously unrewarded color became rewarded.

**Supplementary figure 3. PCA biplot of acoustic song traits.** Blue arrows indicate the contribution (loadings) of each acoustic trait - motif duration, number of motif elements, number of syllables, and similarity to the song model - to the the first two principal components (PC1 and PC2). The direction and length of the arrows reflect the strength and orientation of each trait’s contribution to the axes.

**Supplementary Table 1. Principal component analysis of acoustic song variables.** Variance explained by each principal component in the final PCA based on z-scored acoustic measures (motif duration, number of motif elements, number of song syllables, and similarity to the song model), excluding song bout duration. Values shown are standard deviations, proportion of variance explained, and cumulative variance. PC1 accounted for 73.3% of the total variance.

|  | **PC1** | **PC2** | **PC3** | **PC4** |
| --- | --- | --- | --- | --- |
| Standard Deviation | 1.712 | 0.9351 | 0.37679 | 0.22991 |
| Proportion of variance | 0.7327 | 0.2186 | 0.03549 | 0.01322 |
| Cumulative proportion | 0.7327 | 0.9513 | 0.98678 | 1 |

**Supplementary Table 2. Binomial GLMM results for learning success (success vs. failure to reach criterion.** Binomial generalized linear mixed model (logit link) fitted using glmmTMB. The model included PC1, song bout duration (scaled), and task phase as fixed effects, with bird identity as a random intercept (n = 132 observations from 44 individuals).

| **Predictor** | **Estimate** | **SE** | **z** | **p** |
| --- | --- | --- | --- | --- |
| (Intercept) | 9.655 | 2.082 | 4.638 | < 0.001 |
| PC1 | 0.045 | 0.781 | 0.058 | 0.954 |
| Song bout duration (scaled) | -0.080 | 1.358 | -0.059 | 0.953 |
| Task phase: learning | 1.139 | 1.550 | 0.735 | 0.462 |
| Task phase: reversal | ~0.000 | 1.452 | ~0.000 | 1.000 |
